# Transcranial magnetic stimulation combined with functional near-infrared spectroscopy to elucidate the neurophysiological mechanisms of post-stroke hemiplegia: a systematic review

**DOI:** 10.3389/fneur.2026.1824157

**Published:** 2026-06-10

**Authors:** Yuzhe Zou, Xiangfeng Lai, Qian Liu, Hongmei Zhang, Hui Li, Wei Li, Dingwei He, Liqing Yao, Xue Yang

**Affiliations:** 1 The Second Affiliated Hospital of Kunming Medical University, Kunming, China; 2 School of Rehabilitation, Kunming Medical University, Kunming, China

**Keywords:** fNIRS, hemiplegia, neuromechanism, Stroke, TMS

## Abstract

Patients with hemiplegia after stroke often suffer from motor dysfunction, and their recovery is frequently limited by the imbalanced interhemispheric competition and abnormal neural network reorganization. The combined application of transcranial magnetic stimulation (TMS) and functional near-infrared spectroscopy (fNIRS) has provided a novel closed-loop neuromodulation strategy for the assessment and intervention of post-stroke motor function. This systematic review aims to systematically synthesize clinical research evidence regarding the combined use of TMS and fNIRS in post-stroke motor function recovery, as well as to analyze its neurophysiological mechanisms, clinical efficacy, and methodological quality. By searching databases including PubMed, Web of Science, Embase and Cochrane Library, a total of 9 studies were finally included. The results showed that the TMS-fNIRS combined protocol was safe and feasible, with six of the nine interventional studies reporting statistically significant improvements in motor function (e.g., Fugl-Meyer Assessment scores), while the remaining three did not report clinical outcomes. At the neurophysiological level, effective TMS interventions (such as intermittent theta-burst stimulation (iTBS) or high-frequency repetitive transcranial magnetic stimulation (rTMS) could enhance the activation of the ipsilesional primary motor cortex, facilitate the restoration of interhemispheric balance (with the laterality index shifting toward the ipsilesional side), and improve local and interhemispheric functional connectivity as well as brain network efficiency. Two-thirds of the studies (6/9) found that changes in neurophysiological indicators were significantly correlated with improvements in clinical functions, providing preliminary, hypothesis-generating correlational support for the underlying intervention mechanisms. However, existing studies have limitations including small sample sizes, high protocol heterogeneity, and uneven methodological quality (especially the high risk of bias in non-randomized studies), resulting in a moderate to low level of evidence strength. In conclusion, the combined TMS-fNIRS technology demonstrates the potential to advance stroke rehabilitation toward individualization and closed-loop practice, but current evidence is still in the early stage. Future research needs to conduct large-scale, standardized, algorithm-driven clinical trials to achieve the transition from “proof-of-concept” to “precision therapy”.

## 1 Introduction

### 1.1 Overview of stroke

Stroke remains one of the leading causes of disability and mortality worldwide. Global research data have indicated that stroke was still the second leading cause of death in 2019, accounting for 11.6% of total deaths, as well as the third leading cause of combined death and disability. Between 1990 and 2019, the absolute number of stroke incidents increased by 70.0%, the prevalence of stroke rose by 85.0%, and stroke-related deaths grew by 43.0% (1). According to research data from China, among adults aged 40 years and above, the estimated prevalence, incidence, and mortality of stroke in China in 2020 were 2.6%, 505.2 cases per 100,000 person-years, and 343.4 cases per 100,000 person-years, respectively (2). Beyond the acute mortality and disability, stroke survivors face an elevated risk of long-term complications. Recent large-scale cohort studies have further quantified these risks: the incidence of post-stroke hydrocephalus was reported to be 1.82 per 1,000 person-years, with the adjusted hazard ratio (aHR) highest within 3 years post-stroke (aHR 29.53) and remaining elevated up to 9 years (3). Additionally, dementia is associated with a significantly increased risk of subsequent stroke (aHR 3.19), with the highest risk observed in vascular dementia (aHR 3.58) (4). These findings underscore the substantial and persistent clinical burden of stroke and its sequelae. Motor dysfunction centered on hemiplegia is a common clinical symptom in stroke patients, typically manifesting as muscle weakness, altered muscle tone, abnormal movement patterns, and limited range of motion (5); it affects approximately 55% to 75% of stroke patients, with upper limb impairment occurring in around 85% of them (6). From a pathophysiological perspective, cerebral lesions in stroke patients also disrupt the dynamic balance of brain networks, which is characterized by “imbalanced interhemispheric competition” (7): that is, reduced excitability in the ipsilesional hemisphere and compensatory hyperactivity in the contralesional hemisphere. This theory has been further supported by several studies (8, 9). Consequently, complex and often inefficient reorganization occurs in whole-brain functional connectivity (10). These persistent abnormalities at the neural circuit level constitute the fundamental bottleneck that hinders functional recovery from entering a “plateau stage.” Although task-oriented conventional rehabilitation training remains the cornerstone of clinical practice, a large number of patients find it difficult to break through the plateau in functional recovery. This highlights a fundamental limitation of the current empirical intervention model: there is a lack of both precise assessment tools (e.g., fNIRS) (11) for the underlying neural circuit states that drive functional improvement and targeted regulatory instruments (e.g., TMS) (12). Therefore, the development of a precise neuromodulation technology capable of achieving “assessment-intervention” closed-loop practice has become an urgent need to break through the bottleneck of stroke rehabilitation. This demand provides a critical theoretical and clinical impetus for the technological integration of transcranial magnetic stimulation (TMS) and functional near-infrared spectroscopy (fNIRS) (13).

### 1.2 Development of combined TMS-fNIRS technology

In this paradigm shift, the integration of neuromodulation and neuroimaging techniques plays a pivotal role. Repetitive transcranial magnetic stimulation (rTMS) and its high-efficiency modalities (e.g., intermittent theta-burst stimulation, iTBS) are well-established non-invasive brain stimulation technologies. By stimulating specific cerebral cortical regions to modulate cortical excitability and inter-regional functional connectivity (14–17), they are recognized as powerful tools for inducing therapeutic neuroplasticity. Meanwhile, functional near-infrared spectroscopy (fNIRS), an emerging optical neuroimaging method, non-invasively and real-timely reflects the hemodynamic responses associated with neural activity by monitoring changes in the concentrations of oxygenated hemoglobin (HbO) and deoxygenated hemoglobin (Hb) in the cerebral cortex (18). Compared with functional magnetic resonance imaging (fMRI), fNIRS offers superior motion tolerance, higher temporal resolution, and lower cost (19), making it particularly suitable for synchronous monitoring during rehabilitation tasks, such as working memory tasks (20). Within the combined TMS-fNIRS paradigm, fNIRS has the potential to serve as a feedback sensor in future closed-loop neuromodulation systems. However, it should be noted that true real-time closed-loop TMS-fNIRS has not yet been implemented in the included studies and remains a direction for future research. At present, fNIRS enables immediate and objective quantification of TMS-induced alterations in brain network states (e.g., activation level, connectivity strength) (21). This provides a quantitative basis for dynamically adjusting TMS parameters (including stimulation target, frequency, and intensity) according to an individual's real-time neurophysiological status, thereby laying the technical foundation for achieving truly personalized and precise neural rehabilitation.

### 1.3 Neurophysiological mechanisms of TMS-fNIRS

The combined TMS-fNIRS technology provides a unique window for *in vivo*, non-invasive investigation of neurovascular activity in the cerebral cortex. Its underlying principle is that fNIRS captures hemodynamic responses triggered by neural activity through the mechanism of neurovascular coupling, thus indirectly reflecting cortical excitability and network states (22). A joint fNIRS and TMS study confirmed that cognitive processing speed and neural efficiency could be enhanced through training and neural stimulation (23). Specifically, this technology allows quantitative assessment of the immediate and long-term physiological effects of TMS intervention on specific brain regions and their associated networks, with the main observation dimensions detailed below.

#### 1.3.1 Regional brain activation and cortical excitability

Increased neuronal activity consumes oxygen, but the brain compensates for this demand via vasodilation, leading to a rise in local blood oxygen concentration [[HbO]] (24). fNIRS operates on this principle to monitor changes in the concentrations of oxygenated hemoglobin [Δ[HbO]] and deoxygenated hemoglobin [Δ[Hb]]. A significant increase in Δ[HbO] in the target brain region during or immediately after TMS stimulation, accompanied by task performance, is generally regarded as direct evidence of enhanced local neural activity and vasodilatory response. Early studies demonstrated that following single-pulse TMS stimulation of the primary motor cortex (M1), Hb concentration increased within approximately 3–6 s, peaked at around 6 s, and then returned to baseline levels (25). Relevant research has indicated that high-frequency rTMS or iTBS is also considered to exert excitatory effects; after such stimulation, the Δ[HbO] response in the ipsilesional M1 is enhanced, suggesting that the intervention has successfully elevated cortical excitability in the target area. Conversely, low-frequency inhibitory rTMS (1 Hz) typically results in a decrease in ΔHbO (26).

#### 1.3.2 Interhemispheric balance and inhibition/facilitation

One of the core theories underlying post-stroke motor function recovery is the concept of “imbalanced interhemispheric competition.” TMS-fNIRS quantifies this balance state by calculating the Laterality Index (LI) (21). LI is usually derived from the Δ[HbO] signals of bilateral motor cortices during task performance (27). A shift in the LI value toward the ipsilesional hemisphere (an increase in positive values) indicates that the intervention may have successfully enhanced the involvement of the ipsilesional hemisphere or attenuated the excessive compensation/inhibition of the contralesional hemisphere (28). This constitutes a direct manifestation of the normalization of the interhemispheric inhibition/facilitation balance. Therefore, LI serves as a key neurophysiological indicator for evaluating whether TMS intervention can correct abnormal interhemispheric interactions.

#### 1.3.3 Functional connectivity and brain network reorganization

The high temporal resolution of fNIRS signals enables the calculation of correlations between the time series of different brain regions, which is defined as Functional Connectivity (FC). By analyzing changes in FC strength between the TMS stimulation target and distant brain regions before and after intervention, the trajectory of TMS-induced brain network reorganization can be delineated. For instance, a study combining fNIRS and fMRI found that iTBS could specifically reduce the excessive connectivity between the left dorsolateral prefrontal cortex (dlPFC) and the right insula/operculum, as well as the ipsilateral posterior parietal cortex (29). Another study utilizing fNIRS monitoring revealed that a single session of iTBS stimulation of the left dlPFC could induce significant time-dependent neuromodulatory effects. Ten min post-stimulation, the activation intensity of multiple channels in the prefrontal cortex and the network functional connectivity both reached their peak levels (30). Furthermore, graph theory-based brain network analyses (e.g., clustering coefficient, global efficiency) enable more comprehensive quantification of the information integration and segregation efficiency of the network (31, 32). These analyses provide advanced metrics for evaluating the optimizing effects of TMS on the overall topological properties of the motor-related brain networks.

### 1.4 Objectives of this systematic review

Despite the theoretical attractiveness of the TMS-fNIRS closed-loop paradigm and the emergence of a series of exploratory clinical studies in recent years, evidence accumulation in this field is still in its preliminary stage. Therefore, this systematic review aims to achieve the following objectives:

(1) Comprehensive Evidence Synthesis: Systematically search and summarize clinical studies that apply combined TMS-fNIRS technology to improve post-stroke motor function.
(2) In-depth Element Analysis: Synthesize and analyze the TMS intervention parameters (modalities, targets, treatment courses), fNIRS data acquisition and analysis protocols adopted in each study, and summarize the main findings regarding neurophysiological indicators and clinical functional outcomes.
(3) Evidence Strength Evaluation: Assess the methodological quality and risk of bias of the included studies using standardized tools, and objectively evaluate the overall level and limitations of current evidence.

## 2 Materials and methods

This study was conducted in strict accordance with the guidelines of the Preferred Reporting Items for Systematic Reviews and Meta-Analyses (PRISMA 2020) (33). The study protocol has been registered on the International Prospective Register of Systematic Reviews (PROSPERO), with the registration number CRD420261282558.

### 2.1 Literature search and selection strategy

A systematic literature search was performed across four electronic databases: PubMed, Web of Science, Embase, and the Cochrane Library. The search timeframe covered from the inception of each database to December 31, 2025. The search strategy combined subject headings and free-text terms, with core concepts focusing on “stroke,” “transcranial magnetic stimulation,” and “functional near-infrared spectroscopy.” To ensure comprehensive retrieval, the reference lists of all included studies were manually screened to identify potential additional eligible literature. Inclusion Criteria (PICOS Framework): Population (P): Adult patients (≥18 years old) with post-stroke hemiplegia who had upper or lower limb motor dysfunction and were clinically diagnosed with stroke (e.g., via CT/MRI). No restrictions were imposed on stroke type (ischemic/hemorrhagic), disease duration (acute/subacute/chronic), or severity;

Intervention (I): Studies must incorporate TMS intervention for therapeutic purposes (i.e., repetitive TMS or iTBS, excluding single-pulse TMS used only for assessment). The stimulation target could include the primary motor cortex (M1), other motor-related cortical regions (e.g., supplementary motor area, SMA), or the cerebellum; Comparison (C): No restrictions applied. Comparators could include sham stimulation, conventional rehabilitation, other active interventions, or no comparator; Outcomes (O): Studies must adopt fNIRS as the primary or secondary assessment tool to measure motor function-related cortical neural activity (e.g., blood oxygen response, functional connectivity, network properties), and report clinical functional outcomes; Study Design (S): Clinical studies, including randomized controlled trials (RCTs), non-randomized controlled trials, pre-post self-controlled studies. Exclusion Criteria: Animal experiments and cellular studies; Reviews, meta-analyses, commentaries, conference abstracts, study protocols, and case reports; Studies that only used TMS for intervention without fNIRS assessment, or only used fNIRS for monitoring without combination with TMS intervention; Studies not published in English.

### 2.2 Data extraction

A standardized data extraction form was used to independently extract information from the full texts of included studies by two researchers. The extracted information included the following categories: Basic study information: Author(s), year of publication, country, and study design; Participant characteristics: Sample size, age, gender, disease duration, side of hemiplegia, and baseline clinical scores. Details of intervention measures: TMS parameters: Device model, coil type, stimulation target, stimulation modality (e.g., frequency, intensity expressed as a percentage of resting motor threshold, total number of pulses, single session duration, total treatment course); fNIRS parameters: Device model, number and layout of channels, brain regions covered, light source wavelength, sampling rate, and experimental paradigm. Key study findings, conclusions, and limitations. Any discrepancies during the data extraction process were resolved through discussion between the two researchers.

### 2.3 Outcome measures

Primary Outcomes (Neurophysiological Indicators): fNIRS-derived metrics, including changes in regional cerebral blood oxygen concentration [Δ[HbO], Δ[HbR]], LI, functional connectivity strength (e.g., coherence, phase synchronization), and brain network topological properties (e.g., clustering coefficient, global efficiency). Secondary Outcomes: Clinical motor function scales (e.g., Fugl-Meyer Assessment, Wolf Motor Function Test), balance function scales, and results of correlation analyses between changes in neurophysiological indicators and clinical scores.

### 2.4 Risk of bias assessment

The methodological quality of included studies was independently assessed by two researchers using standardized tools, as detailed below: For randomized controlled trials (RCTs): The Cochrane Risk of Bias Tool 2 (ROB 2) (34) was adopted. Assessments were conducted across five domains: randomization process, deviations from the intended interventions, missing outcome data, measurement of the outcome, and selection of the reported result. For non-randomized controlled trials (e.g., observational studies, pre-post controlled studies): The Risk of Bias in Non-randomized Studies of Interventions (ROBINS-I) (35) was used. Evaluations covered seven domains: confounding variables, measurement of intervention, blinding of outcome assessment, incomplete outcome data, and selective reporting of results. The assessment results were presented in the main text, and any disagreements between the two assessors were resolved through consensus.

### 2.5 Qualitative synthesis approach

Given the substantial heterogeneity in study designs, TMS protocols, fNIRS configurations and outcome measures, meta-analysis was not possible. We therefore followed the Synthesis Without Meta-analysis (SWiM) reporting guideline (36). A harvest plot was constructed to visually summarize the direction of clinical effects, stratified by study design [randomized controlled trials (RCTs) vs. non-randomized controlled trials (non-RCTs)] and TMS modality (excitatory, inhibitory, or combined). Findings were synthesized thematically according to three pre-specified neurophysiological dimensions: (i) regional brain activation and functional connectivity, (ii) interhemispheric balance (laterality index), and (iii) brain network efficiency (graph theory metrics).

## 3 Results

### 3.1 Literature screening process

**Figure 1:**
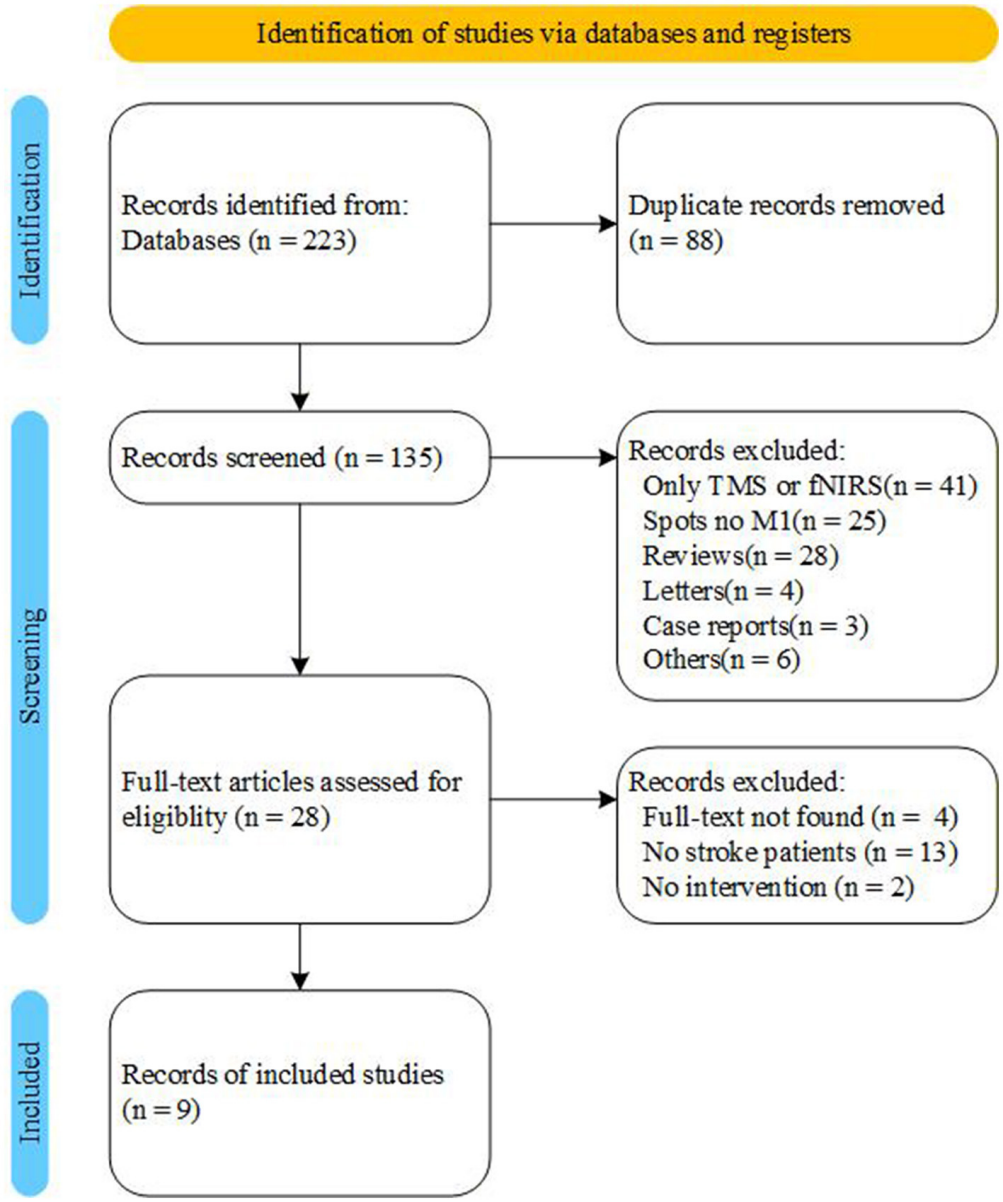
PRISMA flow diagram.

According to the predefined search strategy, a total of 223 relevant records were retrieved. After removing duplicate records, 135 studies remained. A total of 107 studies were excluded after title and abstract screening. Full-text evaluation was conducted on the remaining 28 studies, and finally, nine studies (37–45) met all the inclusion criteria and were included in this systematic review. The detailed screening process, reasons for exclusion, an the number of excluded studies are shown in the PRISMA flow diagram in Figure 1.

### 3.2 Basic characteristics of included studies

The basic characteristics of the nine included studies are detailed in Table 1. All studies were published between 2019 and 2025, of which seven (77.8%) were published after 2021, indicating that this is an emerging field with rapid development. In terms of geographical distribution, eight studies were from China and one from Japan. The study designs included six randomized controlled trials (RCTs) and three non-randomized controlled trials (non-RCTs, including observational and cross-over design studies). The overall sample size was small, ranging from 30 to 68 cases. The average age of patients was between 50 and 70 years old, and the average disease duration varied from several weeks to several years, covering both acute and chronic stroke phases.

**Table 1:** Basic characteristics of the included studies.

Author	Study type	Sample size	Group description	TMS intervention	fNIRS task paradigm	Primary outcome measures
Dai et al. (37), China	RCT	32	iTBS+RT vs. RT	iTBS	Motor task (drinking)	FMA, LI, β values
Tamashiro et al. (38), Japan	Non-RCT	59	Unaffected- vs. affected-hemisphere dominance	LF-rTMS	Motor task (hand movement)	FMA, WMFT, LI
Ni et al. (39), China	RCT	33	Sham, TMS1 (10Hz AH+1Hz UH), TMS2 (10Hz AH)	10 Hz/1 Hz rTMS	Finger-to-nose task	FMA, LI, network parameters
Li et al. (40), China	Non-RCT	30	Stroke group vs. healthy controls	5 Hz rTMS	Resting-state, s-BAT, rTMS-BAT	NIHSS, FMA, effective connectivity
Xie et al. (41), China	RCT	68	LF-rTMS, HF-rTMS, Sham	1 Hz/5 Hz rTMS	Resting-state, during rTMS	FMA, NIHSS, FC, WA
Huo et al. (42), China	Non-RCT	30	Stroke group vs. healthy controls	5 Hz rTMS	Resting-state, s-BAT, rTMS-BAT	FMA, NIHSS, FC, network metrics
Liu et al. (43), China	RCT	52	iTBS group vs. Sham group	iTBS	Resting-state	FMA, MBI, RSFC
Xia et al. (44), China	RCT	31	CB-single, CB-M1, CB-SMA	iTBS	Resting-state, eyes-open/ closed standing	FMA, BBS, COP, FC
Chang et al. (45), China	RCT	51	fNIRS-guided, MEP-guided, Sham	iTBS	Fist clenching task	Brunnstrom stage, FMA, WMFT

RCT, Randomized Controlled Trial; iTBS, Intermittent Theta Burst Stimulation; RT, Robot-assisted Training; rTMS, Repetitive TMS; LF/HF, Low/High Frequency; AH, Affected Hemisphere; UH, Unaffected Hemisphere; M1, Primary Motor Cortex; SMA, Supplementary Motor Area; rTMS, repetitive Transcranial Magnetic Stimulation; s-BAT, Simple Bilateral Arm Training; FMA, Fugl-Meyer Assessment; FC, Functional Connectivity; LI, Laterality Index; WMFT, Wolf Motor Function Test; NIHSS, National Institutes of Health Stroke Scale; MBI, Modified Barthel Index; BBS, Berg Balance Scale; COP, Center of Pressure; RSFC, Resting-State Functional Connectivity; CB, Cerebellum.

### 3.3 Characteristics of TMS intervention protocols

The TMS intervention parameters of each study are specifically summarized in Table 2. Stimulation targets: Most studies (5/9, 55.6%) took the ipsilesional primary motor cortex (M1) as the main stimulation target; 1 study stimulated the contralesional M1, 2 studies stimulated bilateral M1 simultaneously, and another study explored the combined stimulation of the cerebellum and cortical regions (M1 or supplementary motor area, SMA). Stimulation modalities: Intermittent theta-burst stimulation (iTBS, four studies) and high-frequency repetitive transcranial magnetic stimulation (HF-rTMS, ≥5 Hz, two studies) were the main modalities, aiming to excite the ipsilesional cortex; one study used low-frequency rTMS (1 Hz) to inhibit the contralesional hemisphere. Additionally, one study applied a bilateral stimulation protocol combining high-frequency (≥5 Hz) over the ipsilesional M1 and low-frequency (1 Hz) over the contralesional M1. Stimulation parameters: There were significant differences in frequency (1 Hz, 5 Hz, 10 Hz, 50 Hz iTBS), intensity (80%-100% resting motor threshold, RMT), total number of pulses (600–1,200 times), and treatment course (single session to 4 weeks). Some studies combined TMS intervention with specific rehabilitation training (e.g., robot-assisted training, bilateral upper limb tasks).

**Table 2:** TMS parameters.

Author	TMS device	Coil type	Stimulation target	Protocol	Frequency (Hz)	Intensity	Total pulses	Session
Dai et al. (37), China	YRD CCY-I	Figure-of-eight	Affected M1	iTBS	50 Hz	80% RMT	600 pulses	4 weeks
Tamashiro et al. (38), Japan	Mag Pro R30	Figure-of-eight	Unaffected M1	LF-rTMS	1 Hz	90% RMT	1,200 pulses	15 days
Ni et al. (39), China	MagPro R30	Figure-of-eight	Affected M1/Unaffected M1	rTMS	10 Hz/1 Hz	NR	1,200 pulses	4 weeks
Li et al. (40), China	Magneuro	Figure-of-eight	Affected M1	rTMS	5 Hz	90% RMT	1,200 pulses	Single Session
Xie et al. (41), China	NS1000	Figure-of-eight	Unaffected M1/Affected M1	rTMS	1 Hz/5 Hz	100%RMT	600/1,000 pulses	Single Session
Huo et al. (42), China	Magneuro	Figure-of-eight	Affected M1	rTMS	5 Hz	90% RMT	1,200 pulses	Single Session
Liu et al. (43), China	Yiruid	NR	Affected M1	iTBS	50 Hz	80% RMT	600 pulses	8 days
Xia et al. (44), China	NS5000	Figure-of-eight	Cerebellum + M1/SMA	iTBS	50 Hz	80% RMT	600 pulses	Single Session
Chang et al. (45), China	Magstim Rapid2	Figure-of-eight	Affected M1	iTBS	50 Hz	80% RMT	600 pulses	2 weeks

RMT, Resting Motor Threshold; NR, Not Reported.

### 3.4 Characteristics of fNIRS assessment protocols

The technical parameters and analysis protocols of fNIRS are specifically summarized in Table 3. Equipment and coverage: The models of equipment used varied, with the number of channels ranging from 22 to 106. The covered brain regions expanded from focusing on the sensorimotor cortex to multi-brain region networks including the prefrontal cortex and occipital lobe. Task paradigms: They mainly included resting state (5 studies) and block-design task states based on specific movements (e.g., finger opposition, grasping, etc., five studies). The laterality index (LI, three studies), functional connectivity (FC, five studies), and local efficiency, two studies. Analysis indicators: They mainly focused on the laterality index (LI, six studies) for quantifying interhemispheric balance, functional connectivity (FC, six studies), and more advanced brain network topological properties (e.g., clustering coefficient, global efficiency, local efficiency, three studies).

**Table 3:** fNIRS parameters.

Author	fNIRS device	Channels	Wavelength (nm)	Sampling rate (Hz)	Task paradigm	Analysis metrics
Dai et al. (37), China	LIGHTNIRS	22	780, 805, 830	13.33	Motor task (block design)	LI, β values
Tamashiro et al. (38), Japan	FOIRE-3000	49	780, 805, 830	10	Motor task (block design)	LI
Ni et al. (39), China	FORIE-3000	22	NR	NR	Finger-to-nose task	LI, node degree, clustering coefficient
Li et al. (40), China	Multichannel system	NR	NR	10	Rest, s-BAT, rTMS-BAT	Effective connectivity
Xie et al. (41), China	28-channel system	28	740, 850	10	Resting-state, during rTMS	Wavelet amplitude (WA), FC
Huo et al. (42), China	40-channel system	40	NR	10	Resting-state, s-BAT, rTMS-BAT	FC, clustering coefficient, local/global efficiency
Liu et al. (43), China	BS-20000s	106	690, 830	100	Resting-state	RSFC (HbO, HbD, HbT)
Xia et al. (44), China	74-channel system	74	730, 850	11	Resting-state, eyes-open/closed standing	FC, β values
Chang et al. (45), China	Multichannel system	NR	760, 850	6.25	Fist clenching task (block design)	Δ[HbO]

HbO/Oxy-Hb, Oxygenated Hemoglobin; HbD/Deoxy-Hb, Deoxygenated Hemoglobin; HbT, Total Hemoglobin.

### 3.5 Key study findings

**Figure 2:**
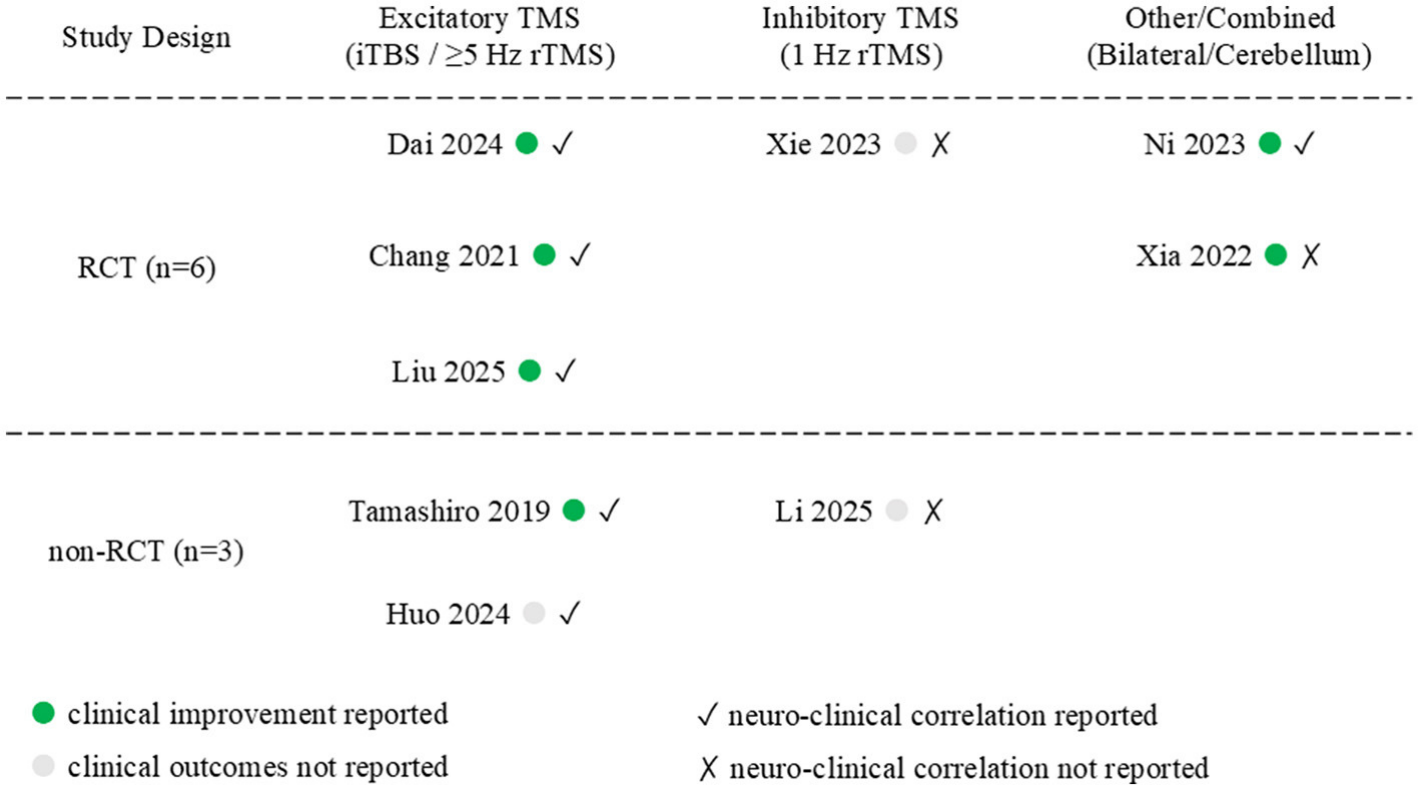
Harvest plot of clinical effects and neuro-clinical correlations among the nine interventional studies. The vertical axis groups studies by design (RCT above, non-RCT below). The horizontal axis categorizes TMS interventions: excitatory (iTBS or ≥5 Hz rTMS), inhibitory (1 Hz rTMS), and other/combined protocols (bilateral or cerebellum-targeted). Green circle: clinical improvement reported. Gray circle: clinical outcomes not reported. 3: neuro-clinical correlation reported. χ: neuro-clinical correlation not reported. The plot follows the SWiM reporting framework and is described in Section 2.5.

The harvest plot (Figure 2) provides an overview of the distribution of clinical improvements and neuro-clinical correlations across different study designs and TMS modalities. The results of the qualitative synthesis of the included studies are summarized in Table 4 and elaborated from the following three dimensions:

**Table 4:** Main results and limitations.

Author	Key neurophysiological findings	Key clinical findings	Neuro-clinical correlations	Main limitations
Dai et al. (37), China	More channels activated in iTBS+RT group; LI shifted positive.	Greater FMA-UE improvement in iTBS+RT group.	ΔFMA-UE positively correlated with Δβ.	Small sample; No long-term follow-up.
Tamashiro et al. (38), Japan	Unaffected: High M1/PMC/SMA activity. Affected: High bilateral (esp. ipsilateral M1/PMC/SMA) activity.	FMA & WMFT improved in both groups.	Baseline LI negatively correlated with FMA/WMFT.	Potential ceiling effect; rTMS not image-guided.
Ni et al. (39), China	Increased affected SMA activation & network parameters post-rTMS.	FMA improved; TMS2 (AH only) > TMS1 (AH+UH).	ΔLI positively correlated with ΔFMA.	Small sample; Subjective FMA; No long-term follow-up.
Li et al. (40), China	rTMS-BAT altered information flow, enhanced interhemispheric coupling.	NR	NR	No sham control; Small sample; Fixed intervention sequence.
Xie et al. (41), China	LF-rTMS induced stronger & sustained contralesional activation & connectivity.	NR	NR	No short channels; Only subcortical strokes included.
Huo et al. (42), China	rTMS-BAT promoted ipsilesional engagement & interhemispheric balance.	NR	Network metrics positively correlated with FMA-UE during tasks.	Cross-sectional; No sham/LF-rTMS control; Small sample.
Liu et al. (43), China	iTBS group had shorter RSFC cycle & earlier peak.	MBI improved more in iTBS group.	Post-treatment MBI correlated with HbD-RSFC.	Small sample; No long-term follow-up; Weak RSFC-scale correlations.
Xia et al. (44), China	CB-SMA group showed prominent network reorganization (inhibitory).	Balance (COP) improved most in CB-SMA group.	NR	Small sample; No FDR correction; No structural MRI; Offline fNIRS.
Chang et al. (45), China	fNIRS hotspot obtainable even if MEP absent; Δ[HbO] correlated with RMT.	Proximal UL function improved more in fNIRS-guided group.	Shorter fNIRS-MEP distance correlated with greater WMFT gain.	Small sample; Heterogeneous lesions; Single-density fNIRS.

#### 3.5.1 Neurophysiological outcomes

Neurophysiological findings were synthesized according to three pre-specified dimensions that align with the SWiM framework (Section 2.5): regional brain activation and functional connectivity, interhemispheric balance, and brain network efficiency. Regional brain activation and functional connectivity. Among the nine interventional studies, three reported significantly increased activation of the ipsilesional M1 or SMA after effective TMS (iTBS or high-frequency rTMS) (37–39). Additionally, five studies demonstrated enhanced FC either within the ipsilesional hemisphere or between bilateral motor-related regions (40–44). For example, Ni et al. observed that high-frequency rTMS over the ipsilesional M1 activated a broader set of cortical areas and shifted the LI toward the ipsilesional side (39); Tamashiro et al. reported that the contralesional hemisphere exhibited high M1/(Premotor Cortex, PMC)/SMA activity, while the ipsilesional hemisphere showed bilateral activation (especially the ipsilesional M1/PMC/SMA) (38). Interhemispheric balance (laterality index). two studies explicitly reported that the LI shifted toward the ipsilesional hemisphere after TMS intervention (38, 39), suggesting that excitatory ipsilesional stimulation (or inhibitory contralesional stimulation) helped correct the pathological interhemispheric imbalance characteristic of post-stroke hemiplegia. Brain network efficiency (graph theory metrics). One study used graph theory-based analyses (clustering coefficient, global efficiency) and found that high-frequency rTMS combined with bilateral arm training improved both local and global network efficiency in patients with chronic stroke (42). Although the evidence for network-level remodeling remains limited (a single study), this finding provides preliminary support for the transition from “node regulation” to whole-brain “network remodeling” (see Section 4.2). In summary, effective TMS interventions consistently increased ipsilesional cortical excitability, restored interhemispheric balance, and in some cases improved functional connectivity and network efficiency, supporting motor recovery after stroke.

#### 3.5.2 Clinical functional outcomes

Of the nine interventional studies, six reported significant improvements in patients' motor function after TMS treatment, mainly reflected by the increase in Fugl-Meyer Assessment of Upper Extremity scores. The remaining three studies did not report clinical outcomes. Some studies also observed positive changes in balance function (Berg Balance Scale) and activities of daily living (Barthel Index). However, the results of direct efficacy comparisons between different TMS protocols were inconsistent. For example, Ni et al. reported that stimulating only the ipsilesional M1 (10 Hz) resulted in greater FMA improvements than the combined protocol of inhibiting the contralesional hemisphere (1 Hz) and exciting the ipsilesional hemisphere (10 Hz) (39); in contrast, Chang et al. found that iTBS navigated by fNIRS “hotspots” was non-inferior to the traditional method navigated by motor evoked potentials (45).

#### 3.5.3 Neuro-clinical correlation outcomes

This is one of the core findings of this review. Two-thirds of the included studies (6/9) reported a significant correlation between changes in neurophysiological indicators and improvements in clinical functionl (37–39, 42, 43, 45). The key correlations included: Prognostic Value of Baseline Status: Specific neurophysiological characteristics before treatment were associated with the degree of subsequent clinical improvement. For example, Tamashiro et al. found a negative correlation between the pre-treatment LI and FMA scores (38), indicating that patients with contralesional hemisphere dominance had poorer motor function. Mechanistic Correlation Value of Intervention-Induced Changes: The magnitude of treatment-induced neurophysiological changes was synchronized with the magnitude of clinical symptom improvement, providing a potential mechanistic explanation for the therapeutic effect. For example, Dai et al. observed a positive correlation between changes in post-treatment brain activation levels (Δβ) and changes in FMA-UE scores (ΔFMA-UE) (37); Ni et al. found that the degree of post-treatment shift in LI toward the ipsilesional side (ΔLI) was positively correlated with ΔFMA (37). Mechanistic Interpretation: These correlational findings provide preliminary, hypothesis-generating support consistent with the hypothesis that TMS may promote motor function recovery by regulating interhemispheric balance and remodeling brain network connectivity. However, given the small sample sizes and the fact that one study did not report correction for multiple comparisons, these findings should be interpreted as exploratory rather than confirmatory.

### 3.6 Risk of bias assessment and methodological quality

Assessments based on standardized tools (ROB 2 and ROBINS-I) showed heterogeneity in the methodological quality of the nine included studies. The main risks of the six randomized controlled trials were concentrated in the “implementation of blinding” link, which might introduce performance bias and detection bias; while the risks in randomization and data completeness were relatively low. The methodological limitations of the three non-randomized controlled trials were more significant. Among them, two studies had “serious” risk of bias, with the main problems including the fixed intervention sequence and the absence of sham control, which made it difficult to isolate the specific treatment effect. In summary, the high risk of bias in the non-RCT part significantly weakened the robustness of the overall evidence, making the inferences about neuro-behavioral causal associations and therapeutic mechanisms drawn from these studies need to be interpreted cautiously.

## 4 Discussion

### 4.1 Summary of core evidence

This systematic review is the first to systematically synthesize clinical studies that combine TMS and fNIRS to explore and promote post-stroke motor function recovery. Through comprehensive analysis of nine studies, we obtained the following core findings: In terms of technical feasibility, the combined TMS-fNIRS protocol is safe and feasible in stroke patients, and six of the nine interventional studies reported positive improvements in motor function (mainly based on FMA) after intervention (37–39, 43–45). At the neurophysiological level, effective interventions (especially excitatory stimulation) are usually accompanied by increased activation of the ipsilesional motor cortex (M1, SMA) (37–39), a shift in the LI toward the ipsilesional side (39), as well as enhanced local and interhemispheric FC and improved brain network efficiency (40–44). Particularly importantly, two-thirds of the included studies (6/9) reported a significant statistical correlation between changes in neurophysiological indicators and improvements in clinical function (37–39, 42, 43, 45), providing preliminary, hypothesis-generating correlational support for the biological effects of the intervention. However, existing studies exhibit high heterogeneity in TMS/fNIRS parameter protocols, sample sizes, and methodological rigor, with an overall high risk of bias, resulting in the current evidence strength remaining at a moderate to low level.

### 4.2 Mechanistic interpretation: from “node regulation” to “network remodeling”

Based on the results of dynamic fNIRS observations, this review provides important empirical evidence for understanding the neural mechanisms by which TMS promotes post-stroke motor function recovery, and also suggests a potential paradigm evolution of understanding rehabilitation mechanisms from local “node regulation” to global “network remodeling.” From the perspective of node regulation, early studies proposed the classic “imbalanced interhemispheric competition” hypothesis (7). This hypothesis holds that after stroke, the inhibitory activity of the ipsilesional hemisphere increases, while the contralesional hemisphere may exhibit compensatory hyperactivity, forming an imbalanced state of interhemispheric inhibition. The results of this review (39) showed that effective TMS intervention enhanced the activation of the ipsilesional motor cortex and shifted the LI toward the ipsilesional side (39), which is consistent with this theory. Meanwhile, several recent studies have indicated that TMS intervention may restore the dominance of the ipsilesional hemisphere by regulating the balance of interhemispheric inhibition (46–49). However, the mechanisms revealed by fNIRS go far beyond changes in local excitability and point to functional reorganization at the whole-brain network level. Modern neural rehabilitation theory increasingly emphasizes that functional recovery depends on the remodeling and reintegration of distributed brain networks (50–52). The enhanced functional connectivity and improved network topological efficiency observed after intervention in this review [e.g., Huo et al. (42)] are highly consistent with the viewpoint of “network neuroplasticity.” However, it should be noted that the evidence for network-level changes currently relies on a limited number of studies, and thus this interpretation remains preliminary. This means that the rehabilitation process is not only about “repairing” the damaged area, but also a process of guiding adaptive plastic changes in the whole-brain network. It also indicates that the role of TMS may promote the formation of more efficient and adaptive motor network patterns by influencing the information flow and dynamic characteristics of the brain network (53–57). Therefore, future assessment and intervention strategies should consider shifting from focusing on “node regulation” of a single target toward exploring “network remodeling” aimed at optimizing overall network coordination.

### 4.3 Toward precision closed-loop regulation: the dual roles of fNIRS

#### 4.3.1 Biomarker role

The evidence from this review supports the clinical translational value of fNIRS indicators in stroke rehabilitation. As a predictive biomarker, fNIRS shows potential in forecasting functional trajectories t based on baseline brain status. This review found that abnormal pre-treatment LI or functional connectivity patterns are associated with poor motor recovery prognosis, consistent with the view that early post-stroke brain network state is a key determinant of functional outcomes. The International Stroke Rehabilitation and Recovery Roundtable has explicitly listed neuroimaging biomarkers as core research tools to identify patient subgroups with different recovery potentials, thereby guiding individualized treatment decisions (58). For example, specific patterns of resting-state functional connectivity have been shown to predict motor learning ability (59) and responsiveness to rehabilitation training (60). Therefore, integrating fNIRS baseline characteristics (such as interhemispheric balance and prefrontal-motor network coupling strength) may help construct prediction models beyond clinical scales. As a monitoring and mechanistic biomarker, fNIRS can objectively and continuously quantify treatment-induced neuroplastic changes. This review confirmed that TMS-induced neurophysiological changes (e.g., ΔLI, ΔFC) are significantly correlated with improvements in clinical function (ΔFMA), suggesting that fNIRS has the potential to serve as a surrogate endpoint to sensitively assess treatment effects in clinical trials (61).

#### 4.3.2 Real-time sensor role

fNIRS also provides a technical bridge for moving from “open-loop” to “closed-loop” neuromodulation. Traditional “open-loop” stimulation uses fixed parameters and cannot adapt to dynamic changes in individual brain states (62). Closed-loop regulation achieves precise guidance of neural activity through an adaptive cycle of “perception-decision-regulation” (63, 64). Due to its good motion tolerance, high temporal resolution, and clinical feasibility, fNIRS is a well-suited feedback sensor for active rehabilitation scenarios (65). In this review, Chang et al. used fNIRS “activation hotspots” for TMS target localization (45), representing an early step toward the individualized “closed-loop” concepts. More advanced closed-loop systems have been demonstrated using EEG signals. Zrenner et al. developed a closed-loop adaptive real-time EEG-TMS system in which a deep neural network learns to automatically identify high- or low-excitability states in the motor cortex and triggers repetitive stimulation at the occurrence of these states, inducing long-term changes in excitability and connectivity (66); similar concepts aiming to decode real-time fNIRS signals into features such as target brain region activation levels and interhemispheric balance indicators, and to dynamically adjust TMS parameters through control algorithms, have not yet been realized but represent a promising future direction. Technical challenges remain, including real-time signal processing, decoding algorithms, and safety verification (67).

In summary, a complete closed-loop TMS regulation system based on real-time fNIRS feedback would form an automated cycle of “perception-decoding-decision-regulation.” Such a system could continuously monitor the brain state and dynamically optimize the stimulation strategy, potentially achieving precise guidance of neuroplasticity.

### 4.4 Critical appraisal of the interhemispheric competition model

The classic “imbalanced interhemispheric competition” model has guided much of the TMS literature in stroke rehabilitation, suggesting that reducing contralesional cortical excitability with low-frequency rTMS can restore interhemispheric balance and improve motor function (38). However, recent evidence has challenged the universality of this model. The bimodal balance-recovery model (68, 69) proposes that the optimal stimulation approach depends critically on the structural reserve of the corticospinal tract (CST). When CST integrity is high (mild-to-moderate impairment), the contralesional hemisphere pathologically inhibits the ipsilesional side, so inhibitory contralesional stimulation is beneficial. When CST integrity is low (severe impairment), the contralesional hemisphere becomes compensatory; thus inhibiting it is harmful, and excitatory ipsilesional or bilateral stimulation is preferred. Empirical support for this model comes from Lin et al. (70), who stratified chronic stroke patients based on the influence of contralesional motor cortices and demonstrated a “bimodal” pattern of interhemispheric inhibition. Recent studies have further confirmed the predictive value of CST integrity for rTMS responsiveness. Wang et al. (71) found that in patients with high CST integrity, low-frequency rTMS over the contralesional M1 yielded better outcomes, whereas in those with low CST integrity, high-frequency rTMS targeting the ipsilesional hemisphere was more effective.

This nuance is highly relevant to the present review, as the included studies applied both excitatory ipsilesional protocols (iTBS or high-frequency rTMS, *n* = 6) and inhibitory contralesional protocols (low-frequency rTMS, *n* = 1), and two studies used a bilateral combination (39, 41). However, none of the studies stratified patients based on CST integrity or used individual biomarkers (e.g., diffusion tensor imaging) to guide the choice of stimulation target or modality. This lack of stratification may explain some of the heterogeneity in clinical responses across studies and highlights a critical gap in the current evidence base.

Future studies should incorporate structural neuroimaging to assess CST reserve and adopt a personalized approach: patients with preserved CST integrity may benefit from contralesional inhibition or ipsilesional excitation, whereas those with severe CST damage may require alternative strategies (e.g., bilateral stimulation, cerebellar stimulation, or neurofeedback-assisted approaches). The bimodal balance-recovery model (68) provides a useful framework for designing future trials and interpreting conflicting results in the literature.

### 4.5 Geographic homogeneity and generalizability

A notable limitation of this systematic review is the geographic concentration of the included studies. After excluding assessment-only studies, eight of the nine interventional studies were conducted in China, with only one from Japan. This homogeneity raises concerns about the generalizability of our findings to other populations, healthcare systems, and cultural contexts. First, China has made substantial research investments in non-invasive neuromodulation and neuroimaging. From 2010 to 2022, the National Natural Science Foundation of China funded 476 neurorehabilitation research projects with a total of 192.38 million RMB (72). Second, cultural differences may influence the applicability of TMS-fNIRS protocols. Wang et al. found that predictors of quality of life for chronic stroke survivors differ between collectivistic (e.g., China) and individualistic cultures, potentially affecting rehabilitation expectations and adherence (73). Third, differences in healthcare systems, such as insurance coverage, rehabilitation accessibility, and post-stroke care pathways, may also affect generalizability. Mulder et al. compared two identically protocolized stroke trials in the Netherlands and Australia and observed significant cross-country differences in patient characteristics and compliance, demonstrating that healthcare system context can influence trial outcomes even under standardized protocols (74). Therefore, while current evidence demonstrates feasibility, international multi-center trials involving diverse populations, healthcare systems, and cultural contexts are urgently needed to validate the generalizability of TMS-fNIRS findings.

### 4.6 Current challenges and future research directions

Despite the broad prospects, this review also reveals severe challenges in this field. Among them is the high heterogeneity of research protocols (see Tables 2, 3). The stimulation targets (ipsilesional M1, contralesional M1, cerebellum, etc.), modalities (iTBS, high/low-frequency rTMS), parameters, and whether to combine rehabilitation training are inconsistent, making it impossible to derive a universal “optimal protocol.” An additional challenge is the wide range of stroke chronicity across the included studies, which spanned from acute (several weeks post-stroke) to chronic (several years) phases. Neural plasticity mechanisms, spontaneous recovery trajectories, and responsiveness to TMS differ substantially across these phases (75). For instance, during the acute and early subacute phases, heightened neuroplasticity may enhance the effects of TMS, whereas in the chronic phase, maladaptive plasticity may have already consolidated, potentially reducing responsiveness or requiring different stimulation parameters (76). The heterogeneous mixing of stroke stages may therefore confound the interpretation of both neurophysiological and clinical outcomes, as none of the included studies stratified patients by time since stroke. Taken together, both protocol heterogeneity and the mixing of stroke chronicity highlight the challenges in standardizing TMS-fNIRS protocols and collectively demonstrate that a “one-size-fits-all” treatment model is unlikely to be optimal (77). Therefore, the focus of future research must undergo a fundamental transformation: From confirming efficacy to exploring prediction models: Large-scale, multi-center, sham-controlled RCTs are urgently needed. The primary goal is not to re-verify “whether it is effective,” but to determine “for whom, when, and with which parameters it is most effective.” Systematic exploration of efficacy prediction models based on multimodal baseline characteristics (including fNIRS network indicators, clinical scales, and imaging features) should be conducted (78). From static protocols to dynamic algorithms: Research should focus on developing and testing adaptive TMS algorithms based on real-time fNIRS feedback. For example, when real-time fNIRS detects that the activation of the ipsilesional M1 reaches a specific threshold or the interhemispheric connectivity tends to be balanced, the algorithm can automatically adjust or terminate the stimulation (79, 80). Promoting technical standardization and in-depth integration: The field needs to reach a consensus on fNIRS data acquisition (such as channel layout, use of short channels to exclude scalp interference), processing workflows, and reporting of key indicators to improve the comparability and reproducibility between studies. Finally, while this review focuses on post-stroke rehabilitation, the closed-loop TMS-fNIRS paradigm may also inform future research into cerebrovascular complications in other high-risk populations. A nationwide 8-year follow-up study in South Korea reported that patients undergoing thoracolumbar spinal fusion had significantly higher risks of subsequent cerebrovascular disease and ischemic heart disease, highlighting the need for improved perioperative vascular risk assessment (81). This finding is consistent with a recent systematic review of spine surgery, which also identified cervical surgery, hypertension, and atrial fibrillation as risk factors for perioperative cerebrovascular accidents (82). Furthermore, a case report has already demonstrated the feasibility of ultra-early navigated rTMS in a patient with perioperative stroke, showing clinical improvement (83). These preliminary findings suggest that neuromodulation protocols developed for stroke recovery might, in the future, be extended to monitor or mitigate cerebrovascular events in surgical patients, though such cross-disciplinary translation remains highly speculative at this stage.

## 5 Limitations

The findings of this systematic review must be interpreted in the context of the following limitations. First, the included studies generally have several inherent methodological limitations, which are also common characteristics of this emerging field: (1) The sample sizes are generally small and no a priori power calculation was performed, resulting in insufficient statistical power, which limits the robustness of subgroup analyses and causal inferences; (2) There is heterogeneity in methodological quality; the difficulty in implementing blinding in RCTs may introduce bias, while non-randomized studies are susceptible to confounding factors; (3) Intervention and assessment protocols lack standardization, TMS and fNIRS parameters vary, and most studies lack long-term follow-up (>6 months), so the durability of neural reorganization and functional improvement cannot be evaluated; (4) Technology integration is still in the initial stage; current studies mainly focus on combined assessment before and after treatment, rather than a true real-time closed-loop feedback system. Second, this review itself has several limitations: (1) The literature search was limited to English databases, which may introduce language bias; (2) Due to the high heterogeneity of the included studies in intervention measures, outcome indicators, and population characteristics, meta-analysis could not be performed to provide quantitative effect sizes, and only qualitative synthesis was possible; (3) This is a rapidly developing research field, and the latest progress after the search cutoff date could not be included.

## 6 Conclusion

The combined application of TMS and fNIRS marks a crucial step toward a neurofeedback-based individualized and precise regulation paradigm in stroke motor rehabilitation. Existing evidence confirms that this strategy can safely induce neurophysiological reorganization associated with functional improvement. However, current studies generally have problems such as small sample sizes, high protocol heterogeneity, and methodological limitations, and the overall evidence is still in the early stage of clinical exploration. Future research needs to achieve a leap from “proof-of-concept” to “efficacy confirmation” and “algorithm-driven.” This relies on: conducting large-scale clinical trials to verify the predictive and monitoring value of fNIRS biomarkers; developing adaptive closed-loop regulation algorithms based on real-time neural feedback; and clarifying the optimal intervention path for different patient subgroups through individualized efficacy prediction models.
