# Correction: Transcranial magnetic stimulation combined with functional near-infrared spectroscopy to elucidate the neurophysiological mechanisms of post-stroke hemiplegia: a systematic review

**DOI:** 10.3389/fneur.2026.1918554

**Published:** 2026-07-23

**Authors:** Yuzhe Zou, Xiangfeng Lai, Qian Liu, Hongmei Zhang, Hui Li, Wei Li, Dingwei He, Liqing Yao, Xue Yang

**Affiliations:** 1 The Second Affiliated Hospital of Kunming Medical University, Kunming, China; 2 School of Rehabilitation, Kunming Medical University, Kunming, China

**Keywords:** fNIRS, hemiplegia, neuromechanism, Stroke, TMS

There was a mistake in Figure 1 as published. The PRISMA flow diagram showed the number of included studies as 11. The correct number of included studies is 9. Two studies were excluded because they did not include TMS intervention. The corrected Figure 1 appears below.

**Figure 1:**
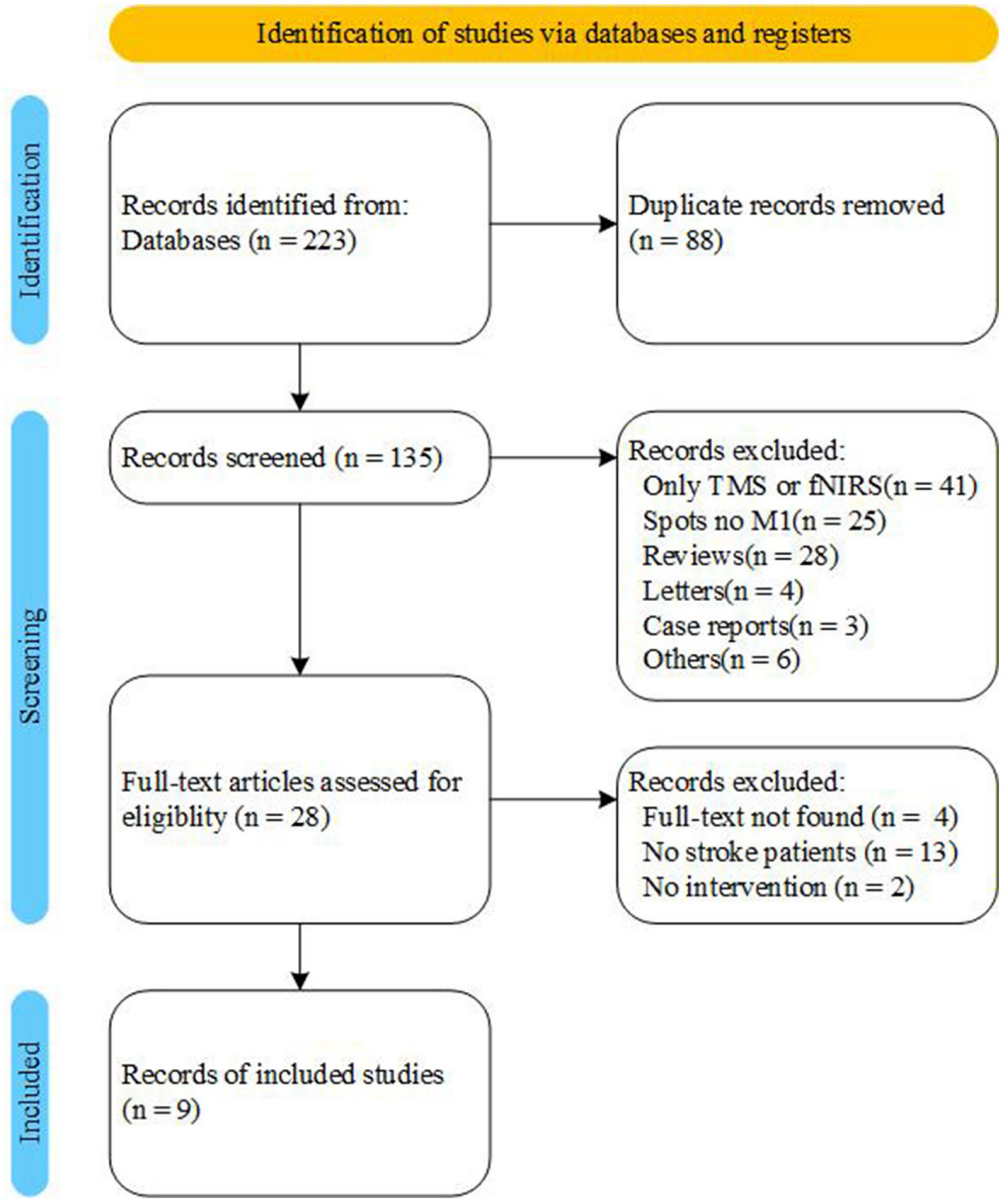
PRISMA flow diagram.

There was a mistake in Figure 2 as published. The study was erroneously labeled as “Tamashiro 2018”. The correct label is “Tamashiro 2019”. The corrected Figure 2 appears below.

**Figure 2:**
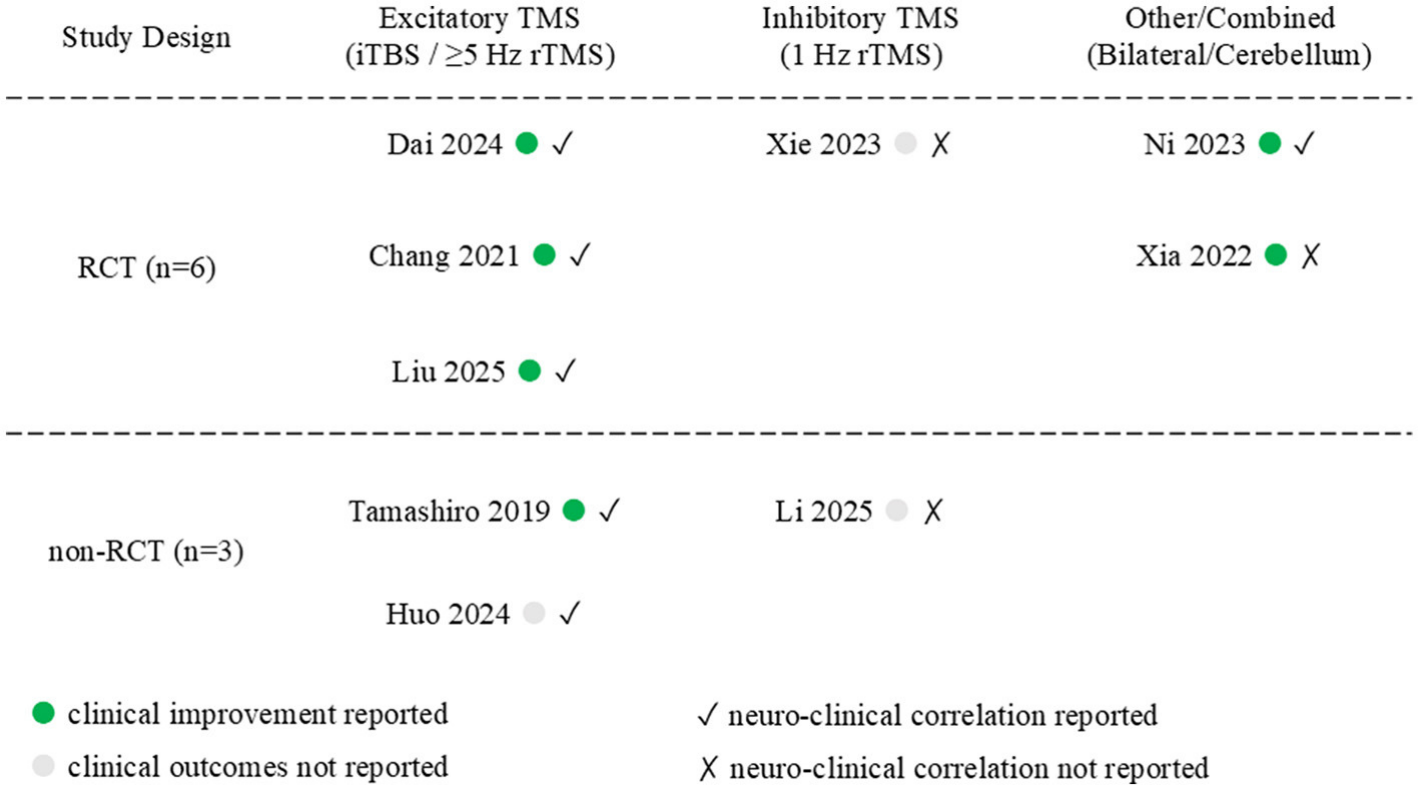
Harvest plot of clinical effects and neuro-clinical correlations among the nine interventional studies. The vertical axis groups studies by design (RCT above, non-RCT below). The horizontal axis categorizes TMS interventions: excitatory (iTBS or ≥5 Hz rTMS), inhibitory (1 Hz rTMS), and other/combined protocols (bilateral or cerebellum-targeted). Green circle: clinical improvement reported. Gray circle: clinical outcomes not reported. 3: neuro-clinical correlation reported. χ: neuro-clinical correlation not reported. The plot follows the SWiM reporting framework and is described in Section 2.5.

There were mistakes in the published text regarding study inclusion numbers, publication years, and task paradigm descriptions.

A correction has been made to section *3.1 Literature screening process*:

“...and finally, nine studies (37–45) met all the inclusion criteria and were included in this systematic review.”

A correction has been made to section *3.2 Basic characteristics of included studies*:

“All studies were published between 2019 and 2025...”

A correction has been made to section *3.4 Characteristics of fNIRS assessment protocols*:

“block-design task states based on specific movements (e.g., finger opposition, grasping, etc., five studies). The laterality index (LI, three studies), functional connectivity (FC, five studies), and local efficiency, two studies.”

A correction has been made to section *3.5 Key study findings*, subsection 3.5.2 Clinical functional outcomes:

“The remaining three studies did not report clinical outcomes.”

The original version of this article has been updated.

